# Employee Perspectives on Onsite Health Clinics in Semiconductor Company in South Korea

**DOI:** 10.3390/ijerph19031433

**Published:** 2022-01-27

**Authors:** Yun-Kyoung Song, Boyoon Choi, Jung Mi Oh, Arim Kwak, Kyungim Kim

**Affiliations:** 1 Research Institute of Pharmaceutical Sciences, College of Pharmacy, Seoul National University, Seoul 08826, Korea; yksong@cu.ac.kr (Y.-K.S.); boyoon1@snu.ac.kr (B.C.); jmoh@snu.ac.kr (J.M.O.); 2 College of Pharmacy, Hayang Campus, Daegu Catholic University, Gyeongsan 38430, Korea; 3 College of Pharmacy, Sejong Campus, Korea University, Sejong 30019, Korea; aarimkwakk@gmail.com; 4 Institute of Pharmaceutical Science, Sejong Campus, Korea University, Sejong 30019, Korea

**Keywords:** employees, workplace, health, onsite clinic, qualitative study

## Abstract

(1) Background: Onsite clinics are increasingly common features of corporate health promotion programs. These clinics allow employers to offer convenient care to employees at their workplaces, which can lead to reduced healthcare expenditure and improved productivity. The objective of this study was to build basic data by qualitatively exploring employees’ experiences and perspectives on onsite clinics in a semiconductor company, as one part of the project to examine and improve the health management system of a large semiconductor company in Korea. (2) Methods: This study adopted the methodology of “Consolidated Criteria for Reporting Qualitative Research” (COREQ-32 checklist). Semi-structured interviews were conducted for this study over a two-month period. For data analysis, a codebook was developed and the constant comparative method was used. (3) Results: Most employees perceived convenience and a sense of belonging as the benefits of onsite clinics, while barriers to the use of onsite clinics included a lack of communication, concerns about confidentiality, and a provider-centered system. Promotion of onsite clinic services and affiliated physicians, employee-centered service provisions, and trust-building in healthcare information privacy were considered necessary to strengthen the role of onsite clinics as a primary care provider in the workplace. (4) Conclusions: The results of this qualitative study help us to gain a better understanding of employees’ perspectives on the onsite clinic’s service and roles.

## 1. Introduction

Health promotion is the process of improving the health of an individual or a community by facilitating better management of health-determining factors [1]. The workplace is an ideal site to promote health because of existing communication channels, culture, and support structure. Worksite health promotion programs (WHPPs) are expanding rapidly and becoming a core strategy for promoting better employee health behaviors and preventing disease, as evidenced by the National Prevention Strategy. It states that workplaces are key “partners in prevention” [2,3]. Several outcome studies found that WHPPs improve employees’ health and decrease sickness-absence costs [4,5,6,7]. Thus, worksite health promotion may be a reasonable approach to increasing workforce productivity and the competitive value of a business. Approximately 26.9 million employees in the Republic of Korea belong to a worksite, accounting for 52% of the total Korean population. Since employees spend an average of 41 h at their workplace every week in Korea, WHPPs play critical health promotion roles at the national level [8,9].

Worldwide, onsite clinics are increasingly common features of WHPPs and have become more prevalent in recent years. Currently, there are a total of 250 onsite clinics in the Republic of Korea, which are continuously increasing from 222 and 241 in 2017 and 2019, respectively [10,11]. In these settings, employers offer one or more medical and wellness services that are delivered by licensed providers to all or a designated portion of the company’s active population and other eligible individuals [12]. Many onsite clinics had started out as occupational health clinics, treating minor injuries and serving employee health and safety needs, eventually expanding to include primary care, chronic disease management, and other areas [13]. Recent studies have reported that onsite clinics reduce healthcare expenditures, establish wellness programs, facilitate trusting relationships among employees, boost productivity, and offer a positive return on the associated financial investment [14].

Clinic services should meet the objectives of the employer’s benefit strategies and employees’ medical needs. The success of the WHPP and onsite clinics depends on a thorough understanding of the factors that motivate employees to actively use the clinic for health promotion as well as the barriers to their involvement [15]. In a survey of employees’ attitudes on a worksite health and wellness clinic in a university, the majority of respondents had a desire to use the clinic [16]. However, few studies have explored what employees feel after using the onsite clinic service. It has been reported that focus group interviews offer a useful means of qualitative data collection for involving users in evaluation of health promotion program and care management and strategy development [17]. Therefore, the aim of this study was to build basic data by qualitatively exploring employees’ experiences and perspectives on onsite clinics in a semiconductor company, as part of a project to examine and improve the health management system of a substantial Korean semiconductor company.

## 2. Materials and Methods

### 2.1. Study Design

This study adopted the methodology of “Consolidated Criteria for Reporting Qualitative Research” (COREQ-32 checklist) (Table S1) [18]. Semi-structured, focus group interviews were conducted for this study.

### 2.2. Study Population

The company that is the background of this study is a large semiconductor company with 5 major domestic business sites and has been operating onsite clinics for each business site. The study population consisted of workers from those business sites. In consideration of the demographic characteristics or diversity of experience, maximum variation purposeful sampling was used for participant recruitment [19]. Potential interviewees were recruited using verbal encouragement from department administration, flyers, e-mail notifications, and word of mouth. Subjects were eligible if they had a minimum of three years of work experience in the company, had received at least one health risk appraisal recommending chronic disease risk management, and had visited the onsite clinic on at least one occasion within the past three years. Then, participants were selected to represent all employees by reflecting characteristics such as gender, age, work type (daytime office worker or shift worker), and the business site they belong to.

The recommended sample size is at least 30–60 people to obtain the rich data needed for qualitative analysis using semi-structured interviews [20]. Therefore, 60 participants were initially invited to participate in the interviews in this study. During the study period, interviews and data analysis were conducted simultaneously, and additional recruitment was performed as needed. The recruitment ended when saturation had been reached in terms of the views expressed, with similar opinions and concepts repeatedly occurring on the study topic. Interviewees gave informed written consent before their participation. The institutional review board of Seoul National University approved this study (IRB No. 1706/002-007, 9 June 2017).

### 2.3. Researchers and Interviewer

A faculty member in pharmacy (the principal investigator, K.K.) with prior training in interviewing and research in health practice led the interviews. No previous personal relationship existed between the interviewer and the participants existed.

### 2.4. Data Collection

The interviews took place between September and November 2017. Each interview group were composed of three to five people, taking account of the affiliated business site, type of work, age, and gender. An interview guide was developed beforehand and reviewed by specialists from the related fields. It included interview questions on experiences and perspectives on the current onsite clinic services (Table 1). We performed a pilot interview with one pharmacy graduate student but did not include it in the data analysis. Each interview took place for about an hour in a separate space inside or outside the workplace. Participants were informed that their names and affiliation would be omitted during the transcription of the recordings to ensure confidentiality.

### 2.5. Data Analysis and Reports

All interviews were audio-recorded and transcribed verbatim by research staff members. To ensure quality, the interviewer checked the accuracy of the interview transcripts.

For the qualitative thematic analysis, this study developed a codebook as recommended by DeCuir-Gunby et al. [21], utilizing the dualistic technique of an inductive and deductive approach [22]. First, initial codes with definitions and examples were deductively created through the initial analysis of the literature and a preliminary scan of the raw interview data by the two primary researchers (Y.K.S. and K.K.). This was then repeated again to determine whether initial codes needed to be changed, or further codes needed to be added. Examples of each code continued to be reviewed and moved until agreement between the researchers as to what determined sufficient demonstration of a true representation of a theme became evident. Then, superior codes and themes were determined and summarized in a codebook. For data analysis, once the codebook was in a draft form, the two primary researchers independently applied a template of codes to all interview transcripts and inductively identified emergent codes and themes. Using the constant comparative method, prior interviews coded were constantly re-analyzed in light of codes that emerged in later analyses, and the codebook was refined. This iterative categorization and conceptualization ensures mutual independence between categories. Throughout the process, any disagreement was resolved either by discussion between the two primary re-searchers, or by considering the opinion of additional researchers (B.C. and J.M.O.) to reach consent.

For reporting, the original language was translated into English by an independent bilingual translator. For validation purposes, the data were double-checked by back-translating English to Korean. The reference at the end of each quotation indicates the participant number followed by the paragraph numbers where the quotation occurs in the transcript, for example, “Participant #3, 109–111”.

## 3. Results

A total of 72 employees via 18 interview groups participated in this study. Table 2 presents a summary of the participant characteristics. Participants’ mean age was 34.2 years old with a standard deviation of 5.1 years. There was an equal proportion of men and women among the participants. As for the job category, 66.7% of the total participants were daytime office workers, and 33.3% performed shift work. Focus group interviews lasted between 48 and 75 min per group, with a mean of 59 min.

Three main themes (perceived benefit, role, and barrier) and nine codes emerged from analyzing the participant interviews (Table 3).

### 3.1. Perceived Benefit

For the theme “perceived benefits to using onsite clinics”, participant thoughts fell into two main categories: (1) convenience and (2) sense of belonging.

#### 3.1.1. Convenience

High convenience was the most frequently mentioned positive factor of using onsite clinics, mentioned by approximately 58% of all participants (*n* = 42). The participants believed that the geographical proximity of onsite clinics was a significant advantage, making visits easier and less restricted by time.

“I would need to take my work hours off to go to an outside hospital. It is easier and faster to go to an onsite clinic since it is always available within our workplace.”

(Participant #7, 134, 135)

“It’s close, so I frequently use an onsite clinic. I prefer the onsite clinics over outside ones since the latter would require me to go out during my work hours. It’s a little cumbersome.”

(Participant #31, 48–50)

#### 3.1.2. Sense of Belonging

Participants responded that they felt respected as an employee and felt a sense of belonging when using onsite clinics and answered that this was a positive factor promoting their use. These feelings were linked to the expectation that the onsite clinic would consistently manage their health and to the trust in onsite clinics. Participants described feeling free of any psychological barriers and comfortable using onsite clinics, resulting from this sense of belonging.

“In outside clinics or university hospitals, physicians tend to be authoritative. At the onsite clinics, however, I feel respected and cared for as an employee. (…) It feels like the clinic is exclusive to me because they have records of all the treatments and medications I have received since I joined the company.”

(Participant #2, 162–164, 215, 216)

“I definitely feel close to the physicians, something like solidarity?”

(Participant #1, 175)

“I don’t think there were any difficulties or inconveniences. People around me have no problem visiting the onsite clinics freely.”

(Participant #50, 411, 412)

### 3.2. Perceived Role

The perceived role of onsite clinics, based on participant experiences, included “primary diagnosis,” “acute illness treatment,” and “chronic disease management.” Although most of the employees found it helpful and a positive experience, not all were happy with their experience.

#### 3.2.1. Acute Illness Treatment

Sixty-four percent of all participants (*n* = 46) responded that the role of onsite clinics was to provide a one-time or short-term treatment for acute or mild diseases. Most participants expected onsite clinics to treat minor ailments, such as a cold, indigestion, or burns. They used onsite clinics only after assessing their symptoms independently and deeming they were treatable there.

“I would go to the onsite clinics for something like a cold. (…) My colleagues all seem to believe that the onsite clinics are for something as mild as a cold. They only visit the onsite clinics for a temporary symptom or else they some take time off to go to an outside hospital.”

(Participant #8, 160, 162–164)

“I go to an onsite clinic for something treatable with medication, such as mild otorhinolaryngologic symptoms or indigestion. If I think my symptoms require more professional treatment, then I would not go to an onsite clinic.”

(Participant #46, 234–236)

A significant contributor to these opinions regarding the role of onsite clinics was their old facilities and equipment. More women than men mentioned this factor.

“The medical equipment looks rather old. I think they have been using the same equipment since I joined the company.”

(Participant #13, 333, 334)

#### 3.2.2. Primary Diagnosis

The second most frequently mentioned role of the onsite clinic was for primary diagnosis. Participants liked how they could receive a primary diagnosis from the onsite clinic and be referred to another hospital if necessary. Many participants were referred to outside hospitals while receiving treatment for a mild condition at an onsite clinic. Such experiences led to trust and satisfaction with onsite clinics as primary care providers.

“A piece of advice as simple as ‘you need to go to this hospital because this has to do with this condition’ was very useful to me. I did not know what my condition was and where to visit on my own. I am personally satisfied with the onsite clinics.”

(Participant #12, 211–214)

“I once visited an onsite clinic for throat pain. Since the pain worsened, they told me to go to an outside hospital. I ended up visiting a regular hospital last year to get it treated. I think the onsite clinics provide a good primary medical care.”

(Participant #58, 232–234)

#### 3.2.3. Chronic Disease Management

All participants received a health risk appraisal recommending chronic disease risk management at their workplaces. However, only 14% (*n* = 10) believed onsite clinics can prevent and manage chronic diseases. Those who believed onsite clinics played a role in chronic disease prevention and management did so due to the quality care and positive support they received from the clinic related to chronic disease. For example, they managed their blood pressure or glucose levels through the onsite clinic, so they believed onsite clinics effectively managed chronic diseases.

“I used to think the onsite clinic was just part of the company, but after receiving chronic disease risk management and consultations, I could tell that they cared about my health. (…) I’ve come to trust the onsite clinics since then. Now, I regularly go for a consultation and blood test every three months.”

(Participant #59, 155, 156, 161–163)

Some participants mentioned the nurse’s office in schools or the medical office of an army as an analogy to onsite clinics and believed that onsite clinics are incapable of providing continuous treatment for chronic diseases. This opinion was expressed more often by shift workers than office workers.

“I do not believe that the onsite clinics can provide continuous treatment. (…) We all think of it as the medical office of an army.”

(Participant #15, 311, 312)

### 3.3. Perceived Barrier

Participants described a wide range of barriers to using onsite clinics properly: the lack of communication about their services, worries related to confidentiality, and the lack of choice in their provider-centered system.

#### 3.3.1. Lack of Communication

There was a significant discrepancy in participant awareness on the information needed to use the onsite clinics. Some participants were highly familiar with the range of treatments offered, hours of operation, reservation methods, and medical staff at their onsite clinics. Most participants (74%) were utterly unaware. They responded that they were unfamiliar with the services provided by onsite clinics and their medical staff and did not know how to find information about them. Most participants received information about onsite clinics from their colleagues. They commented that the scarcity of information about the medical staff made them skeptical about staff professionalism. The participants mentioned that they have low expectations about and trust toward medication prescriptions and treatments given at onsite clinics since they cannot ascertain the professionalism of the medical staff due to a lack of information on their qualifications and work experience. They agreed that the information on the onsite clinic and the medical staff should be delivered so that the employees could fully recognize it.

“I don’t think there’s any way to obtain objective information. The most I can get is just stories from people who visited the clinic.”

(Participant #6, 430, 431)

“I do not think there’s information available about what treatment the onsite clinic provides or the physicians who work there. (…) We don’t know what backgrounds the physicians come from. While advertising the background of the physician is not necessary, I hope that information can be more accessible. (…) It is unclear what treatments are provided at the onsite clinic. In outside hospitals, they have specialized divisions. This is why I think it might be better to just go to an outside hospital that can treat my symptoms. (…) If there are two physicians at an onsite clinic, it’s not like each of them has special expertise. I also don’t know much about them, so I feel afraid and reluctant to go to an onsite clinic.”

(Participant #25, 227–230, 262–264)

#### 3.3.2. Confidentiality

Thirty-five percent of all participants (*n* = 25) mentioned that feeling psychologically burdened about the possibility of having their healthcare information disclosed to the company or their colleagues after using an onsite clinic reduces their willingness to use the onsite clinic and the value of the onsite clinic. This psychological burden was common, felt by participants visiting an onsite clinic to treat an acute or mild condition and those visiting for chronic disease management. Participants believed that having their healthcare information disclosed to their company would negatively affect their work. They expressed feeling psychologically burdened by this and felt negatively toward onsite clinics. They also expressed concerns about having their healthcare information disclosed to their colleagues.

“I just go to an outside hospital because I don’t want people around me talking about my sickness. Some people intentionally avoid leaving any records of their medical history in the company.”.

(Participant #45, 525–527)

“I want to keep my job as long as I can. I felt that using the onsite clinic would make people think I’m a sick person, and that would interfere with my career. (…) It’s not like people denounce me for going to the clinic, but I just feel my self-esteem draining and feel withdrawn, and the stress would, in turn, make me even sicker.”

(Participant #14, 596–598)

#### 3.3.3. Provider-Centered System

Participants responded that the provider-centered management of onsite clinics decreases their willingness to use onsite clinics. The participants felt inconvenienced by onsite clinics providing universal medical services to patients regardless of gender, occupational group (office workers or shift workers), and treatment purpose (acute mild diseases, primary care, or chronic disease management). Furthermore, patients had no way of choosing their physicians. Eleven out of 72 (15%) mentioned that the onsite clinics were either relatively far from their worksite or only operational during the daytime. Shift workers expressed this opinion more frequently. Shift workers explained the difficulty of using onsite clinics for night shift workers since the onsite clinics are only open during the daytime.

“(At our business site) there is a factory that runs for 24 h with three shifts. Onsite clinics are only open from 8 a.m. to 5 p.m.”

(Participant #32, 260–262)

“Since patients are randomly taken to the examination rooms in the order that they arrive, some employees, such as young female employees, may feel uncomfortable when they see a male doctor, especially if they are visiting for a female-specific condition. The clinic doesn’t ask patients how they feel about this, nor does it care.”

(Participant #26, 241–244)

### 3.4. Suggestions

After discussing the research questions, the focus group facilitator asked for suggestions on improvements to the onsite clinic services. Common responses included coordination, communication, and accessibility. The most mentioned suggestion was sharing more basic information about onsite clinics and their activities. Participants said that implementing and expanding a system that continuously informs employees about the news related to onsite clinic management and medical staff/onsite clinic activities would be a significant improvement. They believed that such a system would remove negative preconceptions about onsite clinics and increase trust toward them. Participants also suggested that onsite clinic services should be accessed and improved in conjunction with the entire WHPP. Participants responded that onsite clinics must establish connectivity with related departments (e.g., administrative departments, restaurants, fitness centers, etc.) and provide comprehensive care instead of managing and preventing chronic disease on their own. Additionally, they suggested methods to increase accessibility to onsite clinics, such as improving a reservation system for employees who work far from onsite clinics and investigating and providing the medical services needed by employees.

“Wouldn’t letting employees know that the clinic is doing some specific activities help gain trust among the employees?”

(Participant #2, 604, 605)

“It’s understandable that such misunderstanding exists since not many people are aware of the hard work that the medical staff commit themselves to the onsite clinic. Activities, like advertising, are needed to get rid of the misunderstanding, but currently, there are none. That’s why employees do not feel that the medical staff are working hard.”

(Participant #36, 171–173)

“They are all separate from one another. Workplace cafeteria, onsite clinics, fitness centers.… They all have different administrative divisions that are not connected. To manage chronic diseases, treatments, exercise, and diet should all go together.”

(Participant #43, 483, 488, 489)

## 4. Discussion

Managing employee health and preventing diseases is critical for the health and welfare of workers and the economic and competitive values of companies and countries [4,5,6,7,23]. As part of a WHPP, onsite clinic activities are the most fundamental and essential component of a company’s health management model [12]. In this study, we explored employees’ experiences and perspectives on onsite clinics through 18 focus group interviews among 72 employees of a single semiconductor company and found that while most employees perceived convenience and a sense of belonging after using the onsite clinic service, a lack of communication, concerns about confidentiality and a provider-centered system were found to need improvement to improve the quality of the onsite clinic service and promote its use among the workers.

All interviewees had received health risk appraisals recommending chronic disease risk management in a health assessment and visited an onsite clinic. Interviewees in their 20s to 40s participated in the interviews, and the mean age of the interviewees was 34 years. Since more than 90% of all the employees of the company that was the background of this study were in their 20s to 40s, the age range of the interviewees was deemed suitable [24,25]. Moreover, a previous study reported significantly lower levels of commitment to health-promoting behaviors among employees in their 30s and 40s compared to those in their 50s and 60s. As most employees in their 30s and 40s spend most of their time at work, it is highly critical to listen to the opinions of these age groups on workplace health management [26].

The perceived benefits of onsite clinics reported by the participants were convenience, in terms of geographical proximity, time, and use of service, and a sense of belonging felt by employees when using the medical services provided by their company. Convenience was previously reported as the main advantage of onsite clinics in a systematic review [27]. A sense of belonging has been reported a key support component for physical health in various populations, including college students [28] and community residents [29]. This study showed that the provision of onsite clinic services strengthened workers’ pride and commitment to some extent, which was reported as one of the outcomes of the well-established WHPP [30].

The factors that interfered with the use of onsite clinics were a lack of information about the provided services and medical staff of onsite clinics, concerns about having personal healthcare information disclosed to the company, and the provider-oriented management system, which is consistent with the literature [31]. Bright et al. [16] also reported that the most common barriers for using the onsite clinic service were lack of motivation and work schedule. Based on the health belief model, which proposes changing an individual’s health-promoting behaviors by identifying the factors influencing them and providing interventions, individuals are highly likely to make positive behavioral changes when the perceived benefits outweigh the perceived barriers [32,33]. Thus, to increase the likelihood of an individual engaging in health-promoting behaviors, it is necessary to satisfy the expectations about the perceived benefits of onsite clinics among employees and reduce the inconvenience and difficulties caused by the perceived barriers.

It is noteworthy that employees were concerned about having their healthcare information disclosed to their company or colleagues while recognizing a sense of belonging and solidarity with medical staff as a benefit of onsite clinics. A trust relationship between healthcare providers and employees is a critical factor in health-promoting behaviors [13]. Having healthcare providers and employees work at the same workplace enhances their trust relationship [34]. Therefore, onsite clinics, which are highly accessible in terms of physical distance and time, will encourage health-promoting behaviors among employees [35]. However, a close relationship between healthcare providers and employees poses a concern around healthcare information disclosure. The issue of healthcare information privacy has been an important topic throughout the development of onsite clinics [36]. In this regard, it is worth noting that in a successful onsite clinic model, personal healthcare information is protected by privacy policy/procedure, and employees are reassured that their private health information is protected from their superiors, managers, and colleagues [14].

Treatment of acute or mild ailments was the most common perceived role of onsite clinics among the interviewees. A few participants believed onsite clinics could manage chronic diseases. This is a phenomenon that can be seen at a rather early stage in terms of the development stage of the onsite clinic. Many onsite clinics started as occupational health clinics, treating minor injuries and serving workplace health and safety needs, but today, they have expanded into primary care services, involving management of chronic diseases, preventive care, and other areas [12]. Onsite clinics are the first place employees visit for health in the workplace and can be said to be the company’s primary care provider [13]. Primary care targets acute and chronic diseases. Chronic diseases and their risk factors can be managed efficiently and effectively through primary care. The major characteristics of primary care, including initial contact, consistent management, comprehensive care, and coordinated care, are all essential factors in managing chronic disease [37]. Such characteristics are related to how a majority of the participants mentioned the general scarcity of knowledge about the onsite clinic services available or information about working physicians that led to skepticism about the quality of treatments and the level of professionalism of physicians at onsite clinics. In particular, it needs to be considered that the age of the employees is young, in their 20s and 40s, and they are familiar with methods to obtain and exchange information easily through various media. Therefore, the production of accurate and sufficient information on onsite clinics and appropriate advertising and marketing strategies to increase employee accessibility to the information would help to build employee trust towards onsite clinics and to improve treatment continuity and the self-management ability of the employees.

In this study, shift workers showed less satisfaction with the accessibility of onsite clinics than office workers. They repeatedly mentioned that the role of onsite clinics is limited to providing treatment for acute or mild ailments. Several studies have already reported that shift workers have poorer health compared to office workers. There are reports associating shiftwork with coronary heart disease, and the risk of cardiovascular disease is 40% higher among shift workers than non-shift workers [38,39]. These results suggest the need to provide employee-centered services, for instance, by implementing an onsite clinic management system that considers the characteristics of shift workers.

It was reported that there were pillars of an effective WHPP such as multilevel leadership, alignment, scope, relevance, quality, accessibility, partnership, and communications [30]. The results of this study showed that onsite clinic services of the company have desirable alignment by providing a sense of belonging and adequate accessibility, but a lack of communication and confidentiality and a provider-centered system were found to be obstacles to the use of the clinics. Therefore, sustained employer engagement and investment in the appropriate scale of clinic service with the participation of middle managers and wellness program managers are necessary to lead multilevel leadership.

This study has the following limitations. First, we included 72 employees from a single semiconductor company, which may not be sufficiently representative of onsite clinics from other companies with different environments or cultures. Nevertheless, this company has long been a leading provider of onsite clinics as part of the WHPP. Regarding the number of participants, we applied a maximum variation purposeful sampling method and the constant comparative method, which ensured a sufficient number of participants to reach a state of saturation. In a similar study in which focus group interviews were conducted on health promotion issues in the past, 8–10 focus groups with approximately 60–80 workers were carried out [40,41]. We believe the results of this study are meaningful in providing a basis for better understanding of the employees’ perspectives on onsite clinics and their services. Second, an inherent limitation of qualitative studies is that researchers can introduce bias during data interpretation. In this study, the researchers pursued objectivity and impartiality while minimizing bias by developing a codebook and using the constant comparative method. Lastly, when assessing the services provided by onsite clinics, it is also important to explore the point of view of an employer or healthcare providers. The employers expect the onsite clinics to contain medical costs, improve productivity, enhance the company’s reputation and increase employees’ pride, trust, and commitment by providing a full range of wellness and primary medical services to employees [30,34]. In future studies, it is necessary to collect opinions regarding onsite clinic management from employers or medical staff and to measure the positive impact of clinics on work productivity for a long period.

## 5. Conclusions

The effectiveness and importance of onsite clinics in worksite health promotion are increasingly emphasized. In this study, employees recognized convenience and a sense of belonging as benefits of onsite clinics. On the other hand, factors that hindered employees from using onsite clinics were the lack of information on services and medical staff of the onsite clinic, concerns about the confidentiality of their medical information, and a provider-oriented management system. The results of this qualitative study provide basic data to help us better understand employees’ perspectives on the onsite clinic and its role. Based on the exploratory results of this study, additional research can be conducted to identify important factors for establishing and activating the role of onsite clinics in the future.

## Figures and Tables

**Table 1:** Interview guide.

Introduction
I want to thank you for taking the time to meet with me today. My name is Kyungim Kim. I am a faculty member at the College of Pharmacy. I would like to talk to you about your experience of using an onsite clinic and its services. The purpose of this focus group interview is to hear your experiences, thoughts, and opinions about the clinic at this company. The interview will last about an hour, and it will be audio-recorded because I do not want to miss any of your comments. I will be taking some notes during the session, but I cannot write fast enough to get it all down. Even though we are on tape, please be sure to speak up, so we do not miss your comments. All responses will be kept confidential and shared only with research team members. We will ensure that any information we include in our report does not identify you as the respondent. There are no right or wrong answers to my questions. Please feel free to share your opinions. Remember, you don’t have to talk if you do not want to, and you may end the interview at any time. Are there any questions about what I have just explained? Are you willing to participate in this interview?
Questions
What types of action/services have you experienced from the onsite clinic? What do you think about the current onsite clinic system? – [If positive] In what ways do you think it is positive? – [If negative] In what ways do you think it is negative? What factors do you think would make it difficult to use? What requirements do you think would be necessary to improve the service?
Closing
Is there anything more you would like to add? Thank you for your time.

**Table 2:** Background information of participants.

Variable	Mean (S.D.) or *N* (%)
Age	34.2 (5.1)
Gender	
Male	36 (50.0)
Female	36 (50.0)
Work type	
Daytime office worker	48 (66.7)
Shift worker	24 (33.3)
Affiliated business site ^a^	
A	14 (19.4)
B	18 (25.0)
C	15 (20.8)
D	15 (20.8)
E	10 (13.9)

^a^ Main process of business site: A and B, wafer fabrication; C, research and development; D and E, test and packaging.

**Table 3:** Thematic categories and definitions.

Theme	Code	Definition
1. Perceived benefit		Positive factors that lead to the use of onsite clinic services.
	1. A. Convenience	A quality or situation that makes employees more comfortable or allows employees easier use of onsite clinic services.
	1. B. Sense of belonging	The sense of a positive and lasting relationship with the company or onsite clinic.
2. Perceived role		Perceived onsite clinic roles after experiencing onsite clinic services.
	2. A. Acute illness treatment	One-time or short-term medical services by a healthcare professional to treat acute or mild conditions.
	2. B. Primary diagnosis	Health care by a medical professional with whom a patient has initial contact and can refer the patient to a qualified specialist.
	2. C. Chronic disease management	Longitudinal health care provided by a medical professional for chronic disease.
3. Perceived barriers		Behavior suppressing factors based on onsite clinic experience.
	3. A. Lack of communication	Lack of information about onsite clinic services and health care professionals.
	3. B. Confidentiality	Concerns about the security of individual health issues.
	3. C. Provider-centered system	Employer- or health care professional-centered approaches to providing healthcare services.

## Data Availability

No new data were created or analyzed in this study. Data sharing is not applicable to this article.

## Supplementary Materials

The following are available online at https://www.mdpi.com/article/10.3390/ijerph19031433/s1, Table S1: Description of study methodology using consolidated criteria for reporting qualitative studies (based on COREQ-32 Checklist).
