# Supplementary material for: Employee Perspectives on Onsite Health Clinics in Semiconductor Company in South Korea
Source: Int J Environ Res Public Health. 2022 Jan 27;19(3):1433. doi: 10.3390/ijerph19031433 (PMC8834681; doi:10.3390/ijerph19031433)
Supplement: Supplementary file 1 [file ijerph-19-01433-s001.zip › ijerph-1475372-supplementary.pdf]

Table S1. Description of Study Methodology Using Consolidated Criteria for Reporting Qualitative Studies (Based on COREQ-32 Checklist).

Item	Description
**Domain 1: Research team and reflexivity**	
**Personal characteristics**	
Interviewer/facilitator	K.K.
Credentials	Ph.D.
Occupation	Faculty (College of Pharmacy)
Gender	Female
Experience and training	Currently researching pharmacy and health practice in a college of pharmacy; former pharmacist with hospital and community pharmacy experience.
Relationship with participants	
Relationship established	No prior relationship existed between the interviewer and the participants.
Participant knowledge of the interviewer	The participants did not know the interviewer before their interview. The interviewer introduced herself and explained the goals of the research before their interview.
Interviewer characteristics	The interviewer is a professor at a college of pharmacy and has conducted healthcare-related research for a long time.
**Domain 2: Study design**	
**Theoretical framework**	
Methodological orientation and theory	The dualistic technique of inductive and deductive approach using a codebook and the constant comparative method.
**Participant selection**	
Sampling	Maximum variation purposeful sampling
Method of approach	Verbal encouragement from department administration, flyers, e-mail notifications, and word of mouth
Sample size	72 employees.
Nonparticipation	No participant withdrew from participating in the study.
**Setting**	
Setting of data collection	Separate space inside or outside the workplace
Presence of nonparticipants	No nonparticipants.
Description of sample	Presented in Table 2.
**Data collection**	
Interview guide	Presented in Table 1. Semi-structured interview guide was written by Y.K.S. and reviewed by K.K.
Repeat interviews	No.
Audio/Visual recording	All in-depth interviews were audio-recorded and transcribed verbatim.
Field notes	Field notes were made during the interviews by A.K. when necessary.
Duration	Interviews took 48–75 minutes per group.
Data saturation	Data were collected until we reached thematic saturation and no new codes/themes emerged.
Transcripts returned	No.
**Domain 3: Analysis and findings**	
**Data analysis**	
Number of data coders	Two (Y.K.S. and K.K.).

Item	Description
Description of coding tree	Initial codes with definitions and examples were deductively created through the initial analysis of the literature and a preliminary scan of the raw interview data by the two primary researchers (Y.K.S. and K.K.). For data analysis, once the codebook was in a draft form, the two primary researchers independently applied a template of codes to all interview transcripts, and inductively identified emergent codes and themes. The codebook was refined iteratively with repeat transcript coding and consultative discussions with the authors to ensure methodological rigor.
Derivation of themes	Themes derived from the data. Y.K.S., K.K., and the other authors discussed theme emergence during the data analysis process.
Software	Excel.
Participant checking	Participants did not seek feedback on the findings.
**Reporting**	
Quotations presented	Yes, including the participant reference number.
Data and findings consistent	Yes.
Clarity of major themes	Benefit<br>Role<br>Barrier
Clarity of minor themes	Benefit<br>Convenience<br>Sense of belonging<br>Role<br>Acute illness treatment<br>Primary diagnosis<br>Chronic disease management<br>Barrier<br>Lack of communication<br>Confidentiality<br>Provider-centered system
